# Predictive Role of Acute Physiology and Chronic Health Evaluation II (APACHE II) in Patients With Peritonitis at the National Hospital Abuja

**DOI:** 10.7759/cureus.58412

**Published:** 2024-04-16

**Authors:** Oladele O Situ, Olawale A Badejo, Usman A Gwaram

**Affiliations:** 1 General Surgery, East Lancashire Hospital National Health Service (NHS) Trust, Blackburn, GBR; 2 General Surgery, National Hospital Abuja, Abuja, NGA; 3 Trauma Surgery, National Hospital Abuja, Abuja, NGA

**Keywords:** outcome, laparotomy, peritonitis, apache ii, predictive

## Abstract

Background: A validated tool may facilitate assessing the severity of peritonitis among surgical patients. This study evaluates the predictive role of Acute Physiology and Chronic Health Evaluation II (APACHE II) in the surgical outcomes of patients managed for peritonitis in Abuja.

Method: This is a prospective study of consecutive adult patients managed for peritonitis by the general surgery unit of National Hospital Abuja (NHA) over a 19-month period (September 2020 through March 2022). Patient characteristics and treatment outcomes were recorded in a structured proforma and analyzed using SPSS Statistics version 25 (IBM Corp., Released 2017; IBM SPSS Statistics for Windows, Version 25.0; Armonk, NY: IBM Corp.). The accuracy, sensitivity, specificity, and threshold of APACHE II were derived from the receiver operating characteristic (ROC) curve analysis and its coordinates.

Results: There were 54 patients with peritonitis during the study period, with a male-to-female ratio of 2.6:1. This study's mortality and morbidity rates were 13.0% and 63.0%, respectively. The APACHE II score at admission was positively correlated with the likelihood of postoperative mortality, morbidity, number of postoperative complications, ICU admission, and length of hospital admission. The average APACHE II score of patients in this study was 7.1±5.2. APACHE II best-predicted mortality by the ROC curve at a threshold point of 9 (sensitivity of 85.7%, specificity of 70.2%, the accuracy of 86.8%, P-value 0.002). At a threshold score of 6, APACHE II was significantly associated with the occurrence of postoperative morbidity (sensitivity of 74.3%, specificity of 73.7%, accuracy of 75.2%, P-value = 0.043).

Conclusions: This study confirms that the APACHE II score at admission can predict the outcome of surgery within the first 30 days post-surgery among adult patients who had peritonitis at NHA.

## Introduction

Despite aggressive treatment, the mortality and morbidity from secondary peritonitis are still unacceptably high [1,2]. Mortality rates of peritonitis in many Nigerian studies range from 2.4% to 26.1% [3,4]. Comparing care outcomes among studies is difficult due to the heterogeneity of the disease and severity of the illness among population groups in published studies.

Several scoring systems have been introduced to determine the severity of peritonitis and standardize comparisons between causes of peritonitis. One of the most commonly used scoring parameters is the Acute Physiology and Chronic Health Evaluation II (APACHE II) scoring system developed by Knaus et al. in 1981 [5]. Other assessment systems include the Mannheim peritonitis index, simplified acute physiology score, sepsis severity score (SSS), multiple organ failure scores (Goris score), Ranson score, Imrie score, sequential organ failure assessment score, etc. [6,7].

Although initially designed to study ICU patients, the presently popularized APACHE II risk assessment tool has been validated in large populations of patients for use outside the ICU with good correlates with outcomes even in patients with peritonitis [8,9]. Also, APACHE II considers important parameters such as age and the effect of cardiovascular derangement in the patient, which earlier assessment tools such as acute physiological score (APS) and SSS did not consider, respectively. It has been recommended by the Surgical Infection Society (SIS) as a measure to grade the severity of infection and predict mortality in trials regarding intraabdominal infections [2]. The accuracy of APACHE II in predicting mortality among various groups of patients with peritonitis ranges between 78 and 98% using the receiver operator characteristic (ROC) curve [10-14].

At the time of this study, no electronically available published data validating the complete APACHE II score with the use of arterial blood gas (ABG) in Nigeria existed. This present study attempts to assess the predictive role of the complete APACHE II regarding 30-day morbidity and mortality in patients operated on for peritonitis.

This article was previously presented as a meeting abstract at the 2024 Association of Surgeons in Training annual scientific conference on March 8-10, 2024.

## Materials and methods

Aim

This study aims to evaluate the correlation between complete APACHE II scores and the outcome of surgery for peritonitis among adult patients admitted at the National Hospital Abuja (NHA), vis-à-vis the predictive value of the APACHE II score in patients with surgical peritonitis.

Methods

The study was a prospective cross-sectional study assessing all consecutive patients admitted and operated on for peritonitis within the NHA. Approval was obtained from the research ethics committee of the NHA with an approval number of NHA/EC/066/2020 and file number EXM/PR/SURG/VOL.19/035. The patients' recruitment and data collection was done over a period of 19 months (September 2020 through March 2022). Participants were followed up for 30 days post-surgery. The primary outcome of interest was postoperative mortality following surgery for acute peritonitis, and the secondary outcome was morbidity, ICU admission, length of admission, and risk of multiple surgeries.

The inclusion criteria for this study were adults aged ≥ 18 years and patients with secondary peritonitis. Excluded from the study were patients aged < 18, patients with primary peritonitis, patients who had laparotomy for hemoperitoneum from blunt abdominal solid organ injury, and patients who were lost to follow-up, discharged themselves against medical advice, or who did not give consent.

Following documented informed consent, patients' demographics, investigation results, postoperative diagnosis, and treatment outcomes were obtained in a structured questionnaire. The recorded values at admission prior to resuscitation were used to calculate the APACHE II score. The ABG was analyzed using the i-STAT® system (Abbott Point of Care, Inc., Princeton, NJ, USA). The CG8+ Abbott i-STAT cartridges were used.

All patients had laparotomies. The contaminants were evacuated, the source of contamination was controlled with appropriate surgery, and the peritoneal cavity was thoroughly irrigated with normal saline until clear.

Data results were analyzed using the SPSS software version 25 (IBM Corp., Released 2017; IBM SPSS Statistics for Windows, Version 25.0; Armonk, NY: IBM Corp.). Quantitative (univariate) variables were expressed as frequencies, means, standard deviations, medians, and ranges as appropriate. Pearson's chi-square test and Fisher's exact test were used for bivariate analysis. The level of significance was set at P < 0.05. Student's t-test (t) and chi-square test (χ2) were used to compare differences in means and determine associations for continuous and categorical variables, respectively. The Analysis of Variance (ANOVA) was used to calculate simultaneous differences between three or more mean values of continuous variables. Spearman’s correlation analysis was done to find the relationship between APACHE II and the outcome of patient care. The positive and negative predictive values were calculated from the 2 x 2 contingency table of the χ2 test.

Patients’ APACHE II scores were grouped into strata: 0-10 points, 11-20 points, and > 21 points (as recommended by the joint working party of the SIS North America and Europe) [2]. Morbidity and mortality rates for the stratified APACHE II scores were calculated, and the predictive ability of APACHE II was determined by ROC curve analysis.

## Results

Fifty-four patients were recruited, of which 39 (72.2%) were male and 15 (27.8%) were female (the M:F ratio is 2.6:1). Fifty-four out of 56 eligible patients were recruited successfully for the study (failure-to-enroll rate of 3.6%). By the 30th day following surgery, seven deaths were recorded, and 33 patients had at least one morbidity, giving this study an overall mortality rate of 13.0% and a morbidity rate of 63.0%.

Table 1 shows the direct relationship between the APACHE II score and the outcome following surgery.

**Table 1:** Correlation analysis between APACHE II and the outcome of treatment * significant at 99% APACHE II: Acute Physiology and Chronic Health Evaluation II; ICU: intensive care unit

Outcome	Spearman’s correlation coefficient (r)	P-value
Mortality	0.430*	0.001
Morbidity	0.419*	0.002
Length of hospital stay (days)	0.461*	0.000
Number of complications	0.502*	0.000
ICU stay (days)	0.410*	0.002
Prolonged admission (days)	0.458*	0.000
Multiple surgeries	0.241	0.079
Residual morbidity at 30^th^ day post-surgery	0.238	0.083

The APACHE II scores of the study respondents ranged from 0 to 27, with a mean score of 7.1±5.2. The mean APACHE II score of non-survivors (13.9±6.6) in this study was significantly higher than the mean APACHE II score of survivors (6.0±4.1) (t = 4.324, P-value = 0.000, at 95% confidence interval).

Patients’ APACHE II scores were regrouped into 0-10, 11-20, and ≥ 20 strata and found to have corresponding mortality rates of 7.1%, 27.3%, and 100%, respectively. This was statistically significant (χ2 = 9.972, P = 0.007). When the ANOVA test was used to compare the mean APACHE II score values of the three APACHE score groups for ICU admission and length of hospital stay, they were statistically significant, as shown in Table 2.

**Table 2:** ANOVA test comparing the mean of the three APACHE II groups for hospital and ICU admission * significant at 95% APACHE II: Acute Physiology and Chronic Health Evaluation II; ANOVA: analysis of variance; ICU: intensive care unit

Variable	Mean±SD (APACHE II score group 0-10)	Mean±SD (APACHE II score group 11-20)	Mean±SD (APACHE II score group ≥21)	F	P-value
Hospital admission (days)	11.4±5.4	16.9±8.3	14±0	3.567	0.035^*^
ICU admission (days)	0.1±0.6	3.1±7.9	13.0±0	8.987	0.000^*^

**Figure 1:**
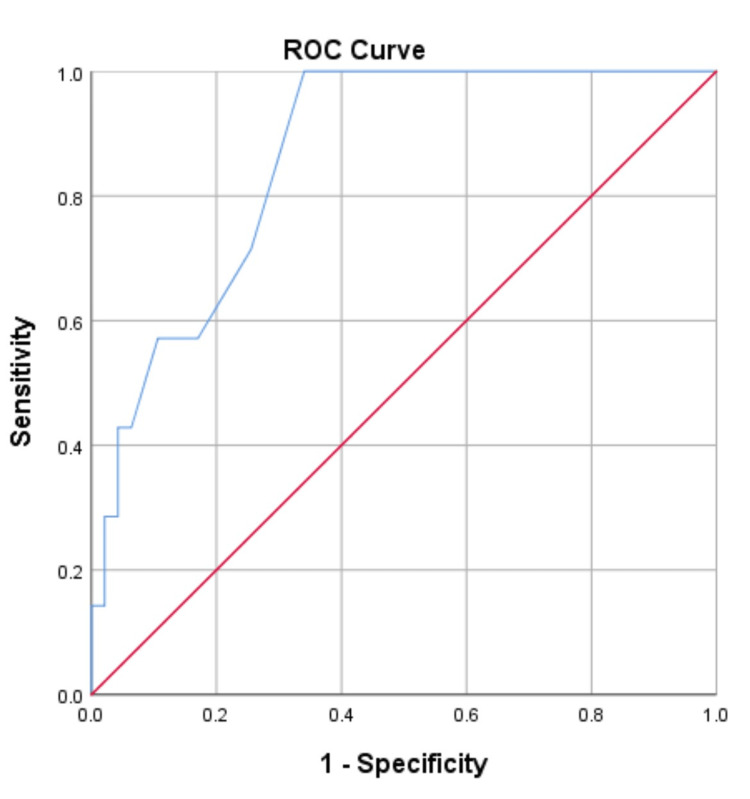
APACHE II ROC curve for mortality AUC = 0.868, P-value = 0.002, sensitivity = 85.7%, specificity = 70.2%, 95% confidence interval. Diagonal segments are produced by ties. AUC: area under curve; APACHE II: Acute Physiology and Chronic Health Evaluation II; ROC: receiver operator characteristic

The ROC curve for mortality (Figure 1) indicates that at the threshold score of nine, the APACHE II model predicted the risk of mortality with an accuracy of 86.8% (represented by the area under the curve), a sensitivity of 85.7%, and a specificity of 70.2% (see coordinates of the curve on Table 3).

**Table 3:** Coordinates of the ROC curve for mortality ROC: receiver operator characteristic

Coordinates of the ROC curve for mortality (positive if greater than or equal to)	Sensitivity	1-specificity
-1.00	1.000	1.000
0.50	1.000	0.936
1.50	1.000	0.894
2.50	1.000	0.809
3.50	1.000	0.660
4.50	1.000	0.596
5.50	1.000	0.511
7.00	1.000	0.319
8.50	0.857	0.298
9.50	0.714	0.255
10.50	0.571	0.170
11.50	0.571	0.106
12.50	0.429	0.064
13.50	0.429	0.021
15.50	0.286	0.021
17.50	0.143	0.021
22.50	0.143	0.000
28.00	0.000	0.000

**Figure 2:**
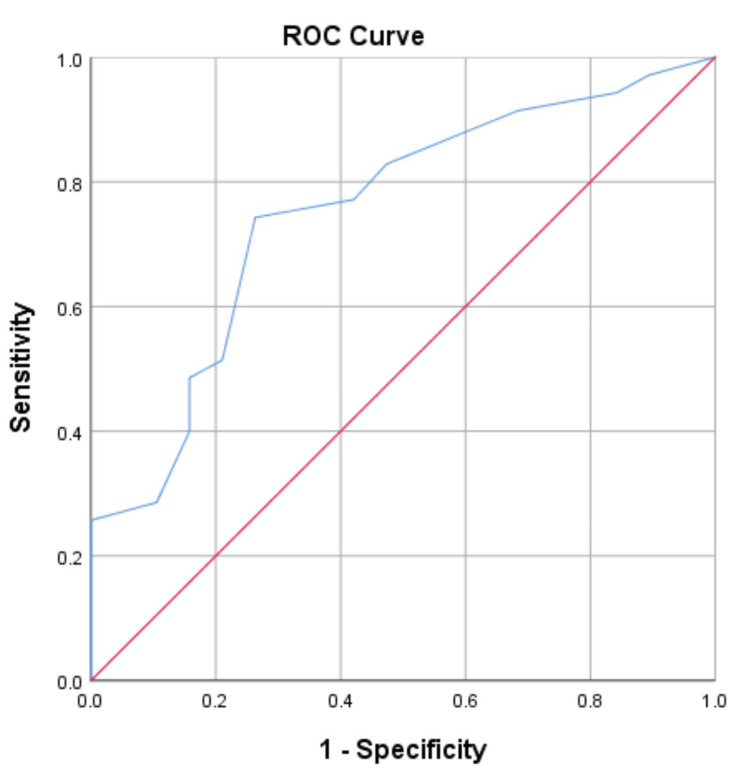
APACHE II ROC curve for morbidity AUC = 0.752, P-value = 0.002, sensitivity = 74.3%, specificity = 73.7%, 95% confidence interval. Diagonal segments are produced by ties. AUC: area under curve; APACHE II: Acute Physiology and Chronic Health Evaluation II; ROC: receiver operator characteristic

The ROC curve for the 30-day postoperative morbidity (Figure 2) and its coordinates (Table 4) show that the APACHE II risk assessment tool predicts the likelihood of morbidity in this study at a cut-off score of six with an accuracy of 75.2% (sensitivity = 74.3%, specificity = 73.7%).

**Table 4:** Coordinates of the ROC curve for morbidity ROC: receiver operator characteristic

Coordinates of the ROC curve for morbidity (positive if greater than or equal to)	Sensitivity	1-specificity
-1.00	1.000	1.000
0.50	0.971	0.895
1.50	0.943	0.842
2.50	0.914	0.684
3.50	0.829	0.474
4.50	0.771	0.421
5.50	0.743	0.263
7.00	0.514	0.211
8.50	0.486	0.158
9.50	0.400	0.158
10.50	0.286	0.105
11.50	0.257	0.000
12.50	0.171	0.000
13.50	0.114	0.000
15.50	0.086	0.000
17.50	0.057	0.000
22.50	0.029	0.000
28.00	0.000	0.000

When all APACHE II scores were dichotomized across a threshold point of nine into two groups, there was a significant association between the primary outcome in this study and the APACHE II groups (χ2 = 5.950, P-value 0.026, PPV 71.4%, NPV 74.4%). The mortality rate in patients in the APACHE II group that do not exceed nine is 5.4%, compared to 29.4% in the patient group with a score greater than nine. Again, the morbidity rate in patients with an APACHE II score group not surpassing six is 53.1%, compared with 81.8% in the patient group with an APACHE II score greater than six (χ2 = 4.707, P-value 0.043, PPV 51.4%, NPV 78.9%).

## Discussion

This study demonstrated that increasing the APACHE II score of the patient at admission had a significant positive relationship with mortality, morbidity, number of complications, duration of ICU admission, length of hospital stay, and risk of prolonged admission. The mean APACHE II score of non-survivors in this study was significantly higher than that of the survivors (P-value = 0.000), and this trend has also been demonstrated in numerous publications [11,13-18].

The mean APACHE II score of 7.1 in this study is in tandem with other research works that strictly assess APACHE II in newly admitted surgical patients with peritonitis [14,15,18-20]. However, the mean APACHE II scores in these studies (including this NHA study) were relatively lower (not above ten) than in large population studies that considered a case-mix of medical and surgical patients admitted into the ICU [11,13,21] (where the mean APACHE II score ranges between 14 and 20). This observation may be because the medically ill patients who require ICU care often have multiple chronic co-morbidities and poor physiologic reserves that significantly raise their APACHE II score calculation compared to surgical patients with peritonitis who are often otherwise well before the acute onset of peritonitis. Therefore, surgeons may not extrapolate the recommendation of studies with case mix (surgical and medical patients) in the ICU to prognosticate newly admitted surgical patients with peritonitis.

A key finding in this study was the identification of a threshold for the APACHE II score beyond which adverse outcomes were likely to occur in patients with peritonitis. These cut-off points have been identified by the ROC curves in this study as having an APACHE score of nine (area under curve (AUC) = 0.868, sensitivity of 85.7%, and specificity of 70.2%) for mortality and six (AUC = 0.752, sensitivity of 74.3%, and specificity of 73.7%) for morbidity. These threshold points were significantly able to classify patients based on the risks of outcome (mortality and morbidity) within the 30-day postoperative period. The mortality rate in patients with an APACHE II score, which is equal to or less than nine, is 5.4% against 29.4% in the patient group with a score above nine (P-value 0.026, PPV 71.4%, NPV 74.4%). The morbidity rate in patients with an APACHE II score that does not exceed six is 53.1%, compared with 81.8% in the patient group with an APACHE II score above six (P-value 0.043, PPV 51.4%, NPV 78.9%). These thresholds have not been earlier described in any known African online study on the role of APACHE II in patients with peritonitis. The ROC curve revealed that the overall predictive accuracy of APACHE II to classify patients that died in this study correctly was 86.8%, and it falls within the general documented accuracy range for APACHE II (80% to 90%) abroad [2,8,10-13,22,23].

The import of these findings is that not only will a surgeon be able to better objectively grade the severity of peritonitis but will also be able to appreciate that some cases would still have poor outcomes despite the aggressiveness of care (i.e., patients with APACHE II scores above nine). Scheduled re-laparotomies may be considered ab initio for these patients with APACHE II scores that exceed nine.

Again, studies [7] (including this NHA study) that tried to find the correlation between APACHE II and the risk of mortality among patients with peritonitis recorded 100% mortality when APACHE II scores exceeded 20. This can also guide the surgeon in the decision-making process and judicious use of medical resources, as studies have questioned the aggressiveness of care in patients with such high APACHE scores [24,25]. However, suppose a surgeon continues with an aggressive surgical line of care for patients with peritonitis who have a very high APACHE II score of ≥ 20. In that case, the surgeon is well informed about the likelihood of the outcome of care. Therefore, when prognostic scoring systems such as APACHE II are properly utilized, the surgeon and the patient (including their caregivers) can better appreciate the severity of the sickness and the possible care outcome. It would improve the quality of care and affect the compassion of care and the allocation of medical resources.

The progressive increase in mortality is seen as the APACHE II score group increases, like the findings of Meakins et al. [26], which confirms evidence for a predictable relationship between APACHE II and mortality as described by Wagner et al. [27]. This study also shows that the APACHE II score grouping of the patient at admission was significantly associated with the risk of having multiple surgeries within the first 30 days of initial surgical intervention for acute peritonitis. This may be explained by the fact that patients with a higher APACHE II score group at admission may have had more derangements in their APS with a higher risk of developing postoperative complications such as organ space SSI and burst abdomen that warranted repeat surgery, as seen in this study.

Progressively increasing the age of patients above 44 years significantly raises their APACHE II score by increasing the ‘age point assessment’ score component of the calculation. Patients younger than 44 have no additional score added to their total APACHE II score. It is therefore not surprising that studies that reported an overall higher mean APACHE II score among their surgical patients with peritonitis [28] recruited older patients (i.e., > 40 years of age) compared with this NHA work (where the mean age of patients was 32.6 years and only 14 out of 54 patients were older than 40 years).

A limitation in the use of APACHE II in this study is that it does not take into account the constant change in the acute physiology score (APS), the duration of deranged APS, and the delay from symptoms onset to surgery. A patient with pre-renal acute kidney injury and an altered level of consciousness from shock may have these parameters corrected with adequate resuscitation before surgery and is expected to have a better postoperative outcome than those patients proceeding to surgery with markedly deranged APS. Literature has shown that delayed presentation is a risk factor for poorer outcomes, independent of APACHE II points at admission [29,30]. Therefore, a one-time APACHE II score at admission may not completely predict the likely outcome of care in all patients, particularly when there are delays before surgery. Multiple APACHE II scores during resuscitation may be a better approach, and the best APACHE II obtained before surgery may best predict the outcome of care post-surgery.

## Conclusions

This study confirms the ability of the full APACHE II score to predict the risk of mortality and morbidity within the first 30 days post-surgery in patients who had peritonitis in Abuja, as shown in other previous studies abroad. APACHE II scores are positively correlated with mortality risk, morbidity, prolonged admission ≥ 14 days, number of postoperative complications, and length of hospital and ICU stay.

Higher APACHE II scores above nine can be regarded as severe peritonitis with a higher risk of mortality. Aggressive care may not translate to survival when the APACHE II score exceeds 20. An APACHE II score above six was associated with a higher risk of postoperative morbidity within 30 days after surgery.
